# Macrophages in bovine term placenta: An ultrastructural and molecular study

**DOI:** 10.1111/rda.13983

**Published:** 2021-07-03

**Authors:** Reyhaneh Hooshmandabbasi, Ali Kazemian, Holm Zerbe, Mariusz P. Kowalewski, Karl Klisch

**Affiliations:** ^1^ Vetsuisse Faculty Institute of Veterinary Anatomy University of Zurich Zurich Switzerland; ^2^ Clinic of Ruminants Ludwig‐Maximilians‐Universität (LMU) Oberschleissheim Germany

**Keywords:** cattle, hofbauer cells, macrophages, polarization, retained foetal membranes

## Abstract

Retention of foetal membranes (RFM) is a major reproductive disorder in dairy cows. An appropriate immune response is important for a physiological expulsion of the foetal membranes at parturition. Our study aims to provide a deeper insight into characteristics of foetal and maternal macrophages in bovine term placenta. We used transmission electron microscopy (TEM), immunohistochemistry and semi‐quantitative RT‐PCR to provide a deeper insight into characteristics of foetal and maternal macrophages in bovine term placenta. Semi‐quantitative RT‐PCR was used to define macrophage polarization in foetal and maternal compartments of normal term placenta. Gene expression of factors involved in M1 polarization [interferon regulatory factor‐5 (*IRF5*), interleukin (*IL*)*‐12A*, *IL12B*] and in M2 polarization (*IL*10) were studied. Ultrastructurally, foetal macrophages showed an irregular shape and large vacuoles, whereas the maternal macrophages were spindle shaped. By immunohistochemistry, macrophages were identified by a strong staining with the lysosomal marker Lysosome‐associated membrane glycoprotein 1 (LAMP‐1), while myofibroblast in the maternal stroma was positive for alpha‐smooth muscle actin. We used the LAMP‐1 marker to compare the density of foetal stromal macrophages in placentas of cows with RFM and in controls, but no statistically significant difference was observed. RT‐PCR showed a higher expression of all studied genes in the maternal compartment of the placenta and generally a higher expression of M1‐, compared to M2‐associated genes. Our results indicated that at parturition placental macrophages predominantly show the pro‐inflammatory M1 polarization. The higher expression of all the target genes in the maternal compartment may denote that maternal macrophages in bovine term placenta are more frequent than foetal macrophages.

## 1 INTRODUCTION

Retention of foetal membranes (RFM) is a common problem in bovine reproduction. It occurs in 3%–12% of parturitions (Esslemont & Kossaibati, 1996; Sheldon, 2004) and negatively affects the subsequent reproductive capability of affected cows and consequently leads to significant economic losses (Dubuc et al., 2011; Kumari et al., 2014). RFM is defined as the failure of expulsion of foetal membranes (FM) within 12–24 hr after delivery of the calf (Beagley et al., 2010). In cases of RFM, the foetal part (cotyledon) of a placentome remains attached to the maternal part (caruncle). To date, although several factors that lead to RFM have been identified (Beagley et al., 2010; Mahnani et al., 2021), the precise biochemical and morphological mechanisms remain elusive. It is already known that in cattle, successful separation and expulsion of FM require orchestration of multiple hormonal, physical and immunological changes (Hansen, 2013; Miyoshi et al., 2002; Streyl et al., 2012). Furthermore, the increased incidence of RFM in cows which share MHC class I antigens with their conceptus highlights the possible role of immunological mechanisms in the release of FM (Benedictus et al., 2015; Joosten et al., 1991).

Macrophages are among the most frequent immune cells in the bovine placenta and have been associated with the detachment of foetal membranes at parturition (Hansen, 2013; Nelli et al., 2019; Streyl et al., 2012). During pregnancy, macrophages gradually and constantly increase in both maternal (Miyoshi & Sawamukai, 2004; Oliveira et al., 2010) and foetal (Schlafer et al., 2000) placental compartments. Moreover, decreased phagocytic activity of the caruncular macrophages has been detected in cows with RFM (Miyoshi et al., 2002). Although several studies have characterized maternal macrophages in the bovine placenta (Mansouri‐Attia et al., 2012; Nelli et al., 2019; Oliveira et al., 2010), foetal macrophages (called Hofbauer cells in human placentas) have not been characterized in detail. Schlafer et al., (2000) showed that the latter cells dramatically increase in number during the last trimester of bovine pregnancy, which reflects a potential role for these cells at parturition. The frequency of these macrophages, however, has not been compared between cows which expelled their foetal membranes normally and those with RFM.

At the foetal–maternal interface, macrophages are implicated in various functions such as regulation of immune cell activities, trophoblast invasion, angiogenesis, embryonic growth and parturition (Erlebacher, 2013). These versatile functions are strongly influenced by the local tissue milieu (Stout & Suttles, 2004). Based on their multiple functions, macrophages have been commonly classified into two subsets, either pro‐inflammatory (M1) or anti‐inflammatory (M2) (Mills et al., 2000). M1 macrophages are typically characterized by high expression of interleukin (*IL*)‐12 and *IL23* and low *IL10* production (Verreck et al., 2004). Conversely, the heterogeneous population of M2 macrophages generally share characteristics such as high *IL10* and low *IL12* and *IL23* expression (Mills et al., 2000; Mosser, 2003) and have been proposed to be crucial regulators of immune responses (Schebesch et al., 1997). *IL12* is a heterodimeric cytokine, which is composed of two different chains, p35 and p40 encoded by *IL12A* and *IL12B* genes respectively. To produce the active form of this cytokine, both genes should be synchronously expressed in the cell (Wolf et al., 1991). While most cell populations are able to produce *IL12A*, the expression of *IL12B* was proposed to be restricted to the cells which produce an active form of *IL12* (D'Andrea et al., 1992; Snijders et al., 1996). It is known that human M1 macrophages have a high expression of interferon regulatory factor 5 (*IRF5*), which induces the transcription of *IL12A* and *IL12B* genes, but prevents the transcription of the *IL10* gene (Krausgruber et al., 2011).

Correct regulation of macrophage polarization is indispensable for a successful pregnancy (Jaiswal et al., 2012; Mor et al., 2011). Similar to human placenta (Cupurdija et al., 2004; Gustafsson et al., 2008), macrophages in cows undergo M2 differentiation over the gestation period (Oliveira et al., 2010). However, as parturition approaches, a shift of polarization towards M1 phenotype takes place in caruncular macrophages which induces inflammatory events, a prerequisite for the detachment of foetal membranes after parturition (Nelli et al., 2019). In cows with RFM, a reduced number of endometrial macrophages along with their differentiation towards M2 polarization has been documented (Nelli et al., 2019).

We hypothesized that at term the cotyledonary (foetal) macrophages differ from the caruncular (maternal) macrophages in morphology and gene expression. To examine this, we compared macrophages in bovine placentomes at term using transmission electron microscopy and immunohistochemistry. In addition, we compared the expression level of *IL10*, *IL12A*, *IL12B* and *IRF5* in the foetal and the maternal part of placentomes. We also used immunohistochemistry for a lysomal marker (LAMP1^+^) to quantify foetal macrophages in placentomes from cows with and without RFM.

## 2 MATERIALS AND METHODS

### 2.1 Subjects and sample collection

Subjects of this study were dairy cows (*Bos taurus*). The origin and number of samples for each objective of the study are listed in Table 1.

**TABLE 1:** Information on the placental samples used in the study

Methods	Placenta samples	Number of animals	Reference/origin of samples
Transmission electron microscopy	Normal release of foetal membranes	5	(Boos et al., 2003)
Confocal microscopy (immunostaining of LAMP1, SMA, PR6)	Normal release of foetal membranes	5	(Greven et al., 2007)
Quantification of foetal macrophages (immunostaining of LAMP 1)	Normal release of foetal membranes and RFM	20 per group	(Braunert, 2012; Streyl et al., 2011)
Gene expression analysis (RT‐PCR)	Caesarean sections (normal release of foetal membranes)	3	Collected at the University of Zurich

Briefly, for electron microscopy, placentomal tissue from cows (*n* = 5) with normal release of the foetal membranes was used. This was material (ethics number: 604i‐42502‐96/907) from one previous study (Boos et al., 2003). From each cow, one placentome was removed within 1 hr after expulsion of the calf and small tissue samples (1 × 1 × 3 mm) were immersed into formaldehyde–glutaraldehyde fixative (Karnovsky, 1965), post‐fixed in 1% (w/v) OsO4 and embedded in EPON^®^ (Serva). Ultrathin sections were prepared from selected regions of interest using an Ultracut^®^ microtome (Reichert‐Jung). The sections were stained with methanolic uranyl acetate and lead citrate (Reynolds, 1963; Stempak & Ward, 1964).

For phenotypic characterization of foetal and maternal macrophages in normal term placenta (*n* = 5), formalin fixed paraffin‐embedded tissues were kindly provided by Prof. Gerhard Schuler (Veterinary Clinic for Obstetrics, Gynecology and Andrology) with ethics number of II25.3‐19c20/15cGI18/14. The preparation process of the paraffin blocks has been previously published (Greven et al., 2007).

The quantification of foetal macrophages was done on material from a larger project (ethics number: 23‐2347‐A‐25‐1‐2009) in which per animal either with RFM (*n* = 20) or normal (*n* = 20), three placental tissue samples were collected at parturition in a systematic random way as described previously (Streyl et al., 2011). Samples were then fixed for 24–48 hr in 3.7% formaldehyde and routinely processed and embedded in paraffin.

In addition, total RNA was extracted from the placentomes collected at routine caesarean sections, which were performed at the Clinic for Animal Reproduction Medicine, University of Zurich (Ethics number ZH 24378). Caesarean sections were carried out in cases of a misfit between foetal size and the maternal pelvic outlet. In all cases, there had been a spontaneous onset of parturition. One placentome was removed immediately after the delivery of the calf. Foetal (cotyledon) and maternal (caruncle) tissues were manually separated. Tissues were stored in RNA later at 4°C for 24 hr and after that transferred to −80°C for long‐term storage. In all cases, the foetal membranes were delivered within 12 hr after the expulsion of the calf.

### 2.2 Transmission electron microscopy (TEM)

Sections were observed with a transmission electron microscope (CM12, Philips) and photographs were taken with a CCD camera (Ultrascan 1000 or Orius 832, Gatan).

### 2.3 Confocal microscopy on three‐color immunofluorescence stained sections

Samples of normal term placenta were subjected to three‐color immunofluorescence using polyclonal rabbit LAMP1‐lysosome Marker (Prod. No. 24170, abcam), monoclonal mouse smooth muscle actin, clone 1A4 (Prod. No. M0851, Dako, Glostrup, Denmark), monoclonal mouse progesterone receptor antibody, clone Alpha PR6 (Prod. No. MA1‐411, Applied Biosystems). In preceding experiments, we tried several established macrophage markers (CD 68, CD 163; CD 206). But these antibodies did not yield a specific signal on the paraffin‐embedded material that was available for our study. Hence, we evaluated another candidate for staining the macrophages, which was a lysosome marker, LAMP‐1 antibody. This antibody showed a strong staining of macrophages in foetal connective tissue when assessed microscopically, and therefore, it was selected as marker for macrophages in this study. The first steps of immunohistochemistry procedure were carried out as described previously (Hooshmandabbasi et al., 2018). After sections were incubated with blocking buffer, they were overlaid with anti‐LAMP1 antibody (1:2500), SMA antibody (1:200) and PR antibody (1:200). As for isotype control, a combination of mouse IgG2a antibody (1:200, Exbio, Vestec, Czech Republic) and rabbit IgG antibody (1:400, Vector Laboratories) was applied. All slides were incubated overnight at 4°C in Shandon's Coverplates. The fluorochrome conjugated secondary antibodies alexa fluor goat anti‐mouse 488 and alexa fluor goat anti‐rabbit 594 (Thermo Fisher Scientific, Rockford, IL, USA) were utilized. They both were diluted 1:200 in a DAPI nucleo‐staining solution (Invitrogen) which had been diluted 1:1000 in IHC buffer. Following 3 × 10 min IHC buffer washing, the slides were taken out of coverplates. They were washed first in 2% formalin (diluted in Aqua distilled water) for 10 min on the shaker and then very carefully with distilled water. Finally, cover slips were mounted using Fluoromount (Sigma, St. Louis, USA). In order to control the specificity of the two mouse antibodies, mono‐staining was carried out for each of them separately. The sections were then analysed using a confocal laser scanning microscope (SP8, Leica).

### 2.4 Immunohistochemistry (IHC)

Immunohistochemistry was employed to demonstrate foetal macrophages located in foetal connective tissue. Immunohistochemical detection was achieved by an indirect immunoperoxidase method as previously described (Hooshmandabbasi et al., 2018). In brief, preparation of the sections for being overlaid with the primary antibody was carried out as described above. After sections were incubated with blocking buffer, they were overlaid with a rabbit polyclonal anti‐LAMP1 antibody ‐Lysosome Marker (ab24170, Abcam) at 1:2,500 dilution, in Shandon's coverplates overnight at 4°C. To dilute the antibody, IHC buffer/0.3% Triton X pH 7.2–7.4 was used. For isotype control and negative control slides, the primary antibody was replaced by non‐immune rabbit IgG (Dako) at the same protein concentration as for the primary antibody or IHC buffer/0.3% Triton respectively.

On the following day, the sections were first washed with IHC buffer/0.3% Triton and then incubated with biotinylated goat anti‐rabbit antibody (1:100, Vector Laboratories) for 30 min at ambient temperature. Thereafter, the signals were boosted using a 30‐min incubation with the Vectastain ABC Kit (Vector Laboratories, Inc.). The immune reactions were then visualized with the DAB+substrate kit (Dako Schweiz AG, Baar, CH). The slides were then counterstained with haematoxylin. Next, ethanol series and xylene were employed for dehydration. Finally, the coverslips were mounted using Histokit (Assistant).

### 2.5 Estimation of the density of foetal macrophages

**FIGURE 1:**
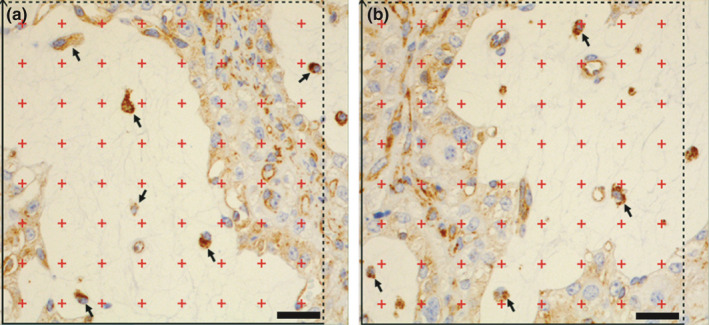
LAMP1 immunohistochemistry of bovine placentomes at parturition of a control (a) and of a cow with RFM (b). The LAMP1‐positive macrophages (arrows) in fetal connective tissue were quantified, using an unbiased counting frame (black lines). The 64 test points (red crosses) were used to determine the fraction of fetal connective tissue. Scale bars = 25 μm

From each animal, three slides were used for the quantification. The entire stained slides were digitized using a digital slide scanner (Nanozoomer HT 2.O, Hamamatsu, Japan), and then, NDPview2 software was used for image acquisition. At a low magnification (× 1.26) regions were selected in a random systematic way. The number of these regions per slide varied dependent on the size of the sections (mean 32.3, minimum 9, maximum 59 regions per slide). These regions were imaged at a higher magnification (×40), which was appropriate for studying the cellular morphology. Subsequently, a grid with 64 test points and an unbiased counting frame was superimposed to each image (Figure 1) and points in the foetal connective tissue were counted. The number of macrophages in the foetal connective tissue was then documented. Cells were counted as macrophages if they had a strong cytoplasmic signal for LAMP1 and a part of the nucleus was visible in the section. Image acquisition and counting was done under blinded condition where the investigator (A.K) did not know the allocation of slides to the groups. The density of foetal macrophages within the foetal connective tissue was estimated (LAMP1^+^ cells/mm^2^ foetal connective tissue) for each animal. For this, the counted macrophages in all three slides were added and divided by the total hits in foetal connective tissue, which represented the reference volume. Finally, the mean values for the RFM‐group were compared to the control group by unpaired two‐tailed Student's *t*‐test. The level of significance was considered as *p* < .05.

### 2.6 RNA isolation, reverse transcription (RT), semi‐quantitative (TaqMan) PCR

Semi‐quantitative real‐time (TaqMan) PCR was performed to compare the expression level of *IL10*, *IL12A*, *IL12B* and *IRF5* genes within the foetal (cotyledon) and maternal (caruncle) parts of bovine placenta (*n* = 3). Samples (0.5 × 0.5 × 1 cm) were immersed in 1 ml ice cold TRIzol (Invitrogen). Total RNA was isolated according to the manufacturer's protocol and previously published protocols (Kowalewski et al., 2006). Quality and concentration of the extracted total RNA were determined with a NanoDrop 2000C spectrophotometer (Applied Biosystems). RNA samples were then treated with RQ1 RNase‐free DNase (Promega) and were reverse transcribed into cDNA using random hexamers as primers along with the other RT reagents (Applied Biosystems). PCR assays were performed in an automated fluorometer (ABI PRISM^®^ 7500 Sequence Detection System, Applied Biosystems) according to previously published method (Kautz et al., 2015; Kowalewski et al., 2006). Briefly, reactions were run in duplicates with Fast Start Universal Probe Master (ROX)^®^ (Roche Diagnostics AG). A non‐template control was performed by using autoclaved water instead of cDNA. Three reference genes (*GAPDH*, *SDHA* and *ACTB*) were used to test the integrity of RNA and to normalize gene expression profiles. As for GAPDH and SDHA, customized bovine‐specific primers and 6‐FAM‐ and TAMRA‐labelled (TaqMan) probes were purchased from Microsynth, Balgach, Switzerland (Table 2). The commercially available IL10, IL12A, IL12B, IRF5 and ACTB TaqMan Gene Expression Assay were ordered from Applied Biosystems (Prod. No. Bt03212724_m1, Bt03213919_m1, Bt03213924_m1, Bt03222257_g1 and Bt03279175_g1 respectively). Data were assumed valid if the cycle threshold (CT) of reference genes for a sample was constant. The results are expressed as the fold change in gene expression over the calibrator. The comparative CT method (ΔΔ CT method) was used for the relative quantification of genes as previously described (Kowalewski et al., 2008) and the sample with the lowest expression was used as a calibrator. To calculate the *IL10/IL12A* and *IL10/IL12B* ratio, the same CT values and calibrators were applied and then relative gene expression values for each pair were divided. As the data did not possess a normal distribution, logarithmic transformations were performed to normalize the final data. Statistical differences in the expression of the target genes between cotyledonary and caruncular parts of the placenta and also the ratio between pair genes were calculated by an unpaired two‐tailed Student's *t*‐test. The expression levels of the four target gene were compared within either foetal or maternal group by a parametric one‐way ANOVA. When P‐value was smaller than 0.05, it was followed by the Tukey–Kramer multiple comparisons post hoc analysis. All analyses were performed with GraphPad 3.06 software (GraphPad Software). The numerical data are shown as geometric means (Xg) ± geometric standard deviation (*SD*). Values of *p* < .05 were considered as statistically significant.

**TABLE 2:** List of customized primers and probe sets used for real‐time (TaqMan) PCR

Gene symbol	NCBI reference sequence	Sequence	
GAPDH	NM_001034034	Forward	5′‐GCG ATA CTC ACT CTT CTA CCT TCG A‐3′
		Reverse	5′‐TCG TAC CAG GAA ATG AGC TTG AC‐3′
		TaqMan probe	5′‐CTG GCA TTG CCC TCA ACG ACC ACT T‐3′
SDHA	NM_174178	Forward	5′‐ATG GAA GGT CTC TGC GCT AT‐3′
		Reverse	5′‐ATG GAC CCG TTC TTC TAT GC‐3′
		TaqMan probe	5′‐ACA GAG CGA TCA CAC CGC GG‐3′

## 3 RESULTS

### 3.1 Maternal and foetal macrophages have different ultrastructural characteristics

**FIGURE 2:**
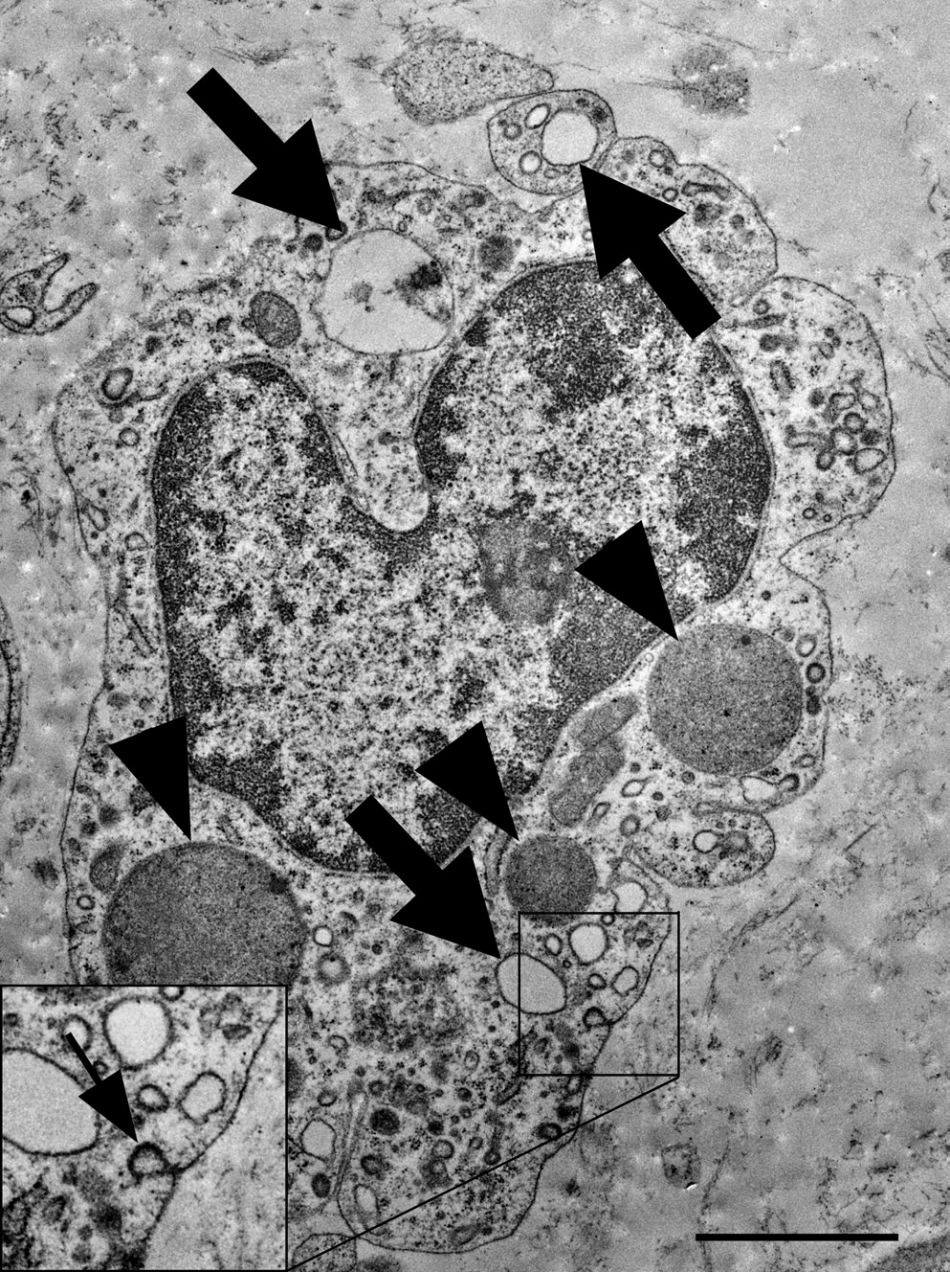
Transmission electron micrograph. One fetal macrophage/Hofbauer Cell is located in the loose stromal tissue of a cotyledonary villus. Several electron‐dense lysosomes are labelled with black arrowheads and empty vacuoles (large arrows) were partially filled with flocculent material. The inset shows a coated vesicle (small arrow) at higher magnification. Scale bar = 2 μm

In transmission electron microscopy, macrophages in the foetal cotyledonary stroma could be easily recognized (Figure 2). These cells had an irregular shape with several processes. One characteristic feature was the abundance of large ‘empty’ vesicles, which were partially filled with flocculent material. Lysosomes with an irregular electron‐dense content were also visible. Coated pits and vesicles were frequently observed.

**FIGURE 3:**
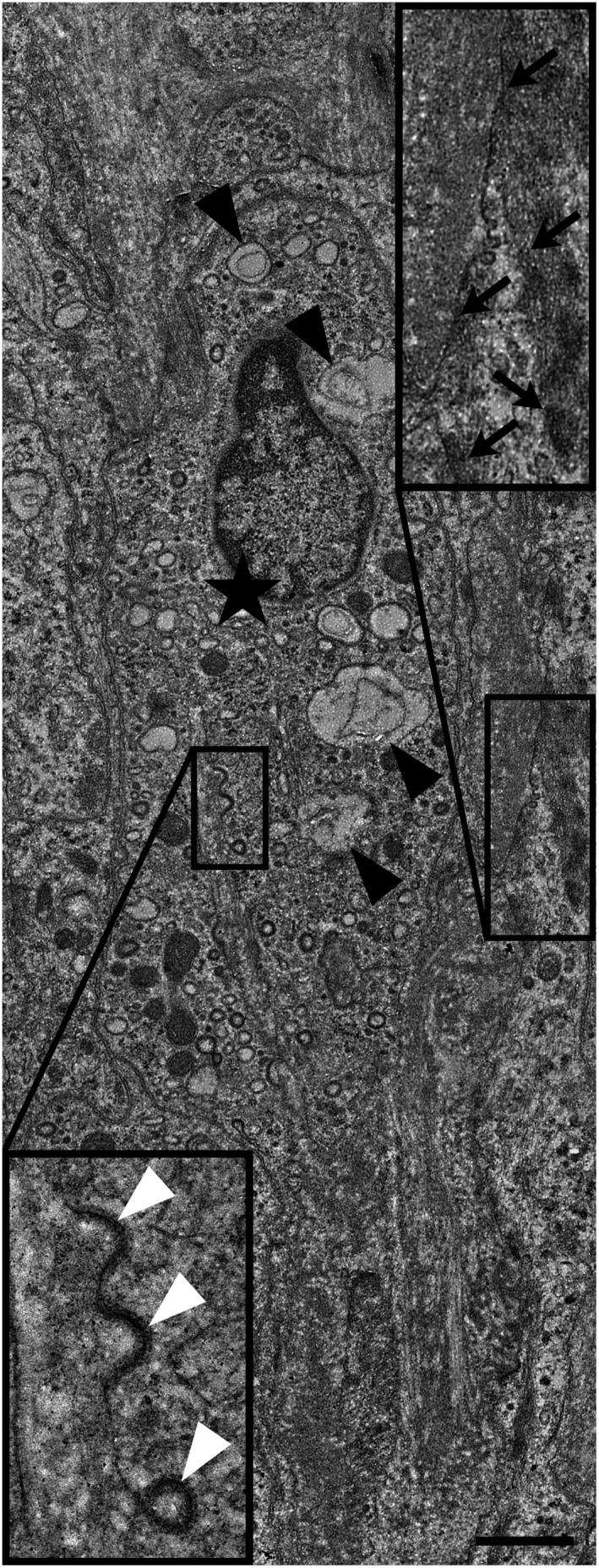
Transmission electron micrograph. Maternal macrophage (star) in a caruncular septum. Several empty vesicles are present and the four largest are labelled with black arrowheads. Coated pits and one coated vesicle (bottom inset, white arrowheads) are frequently observed. One adjacent myofibroblast shows dense bodies and adhesion plaques (arrows in the upper inset). Scale bar = 1 μm

The macrophages in the maternal caruncular stroma showed a different morphology. These cells were elongated spindle shaped with several processes (Figure 3). Coated pits and vesicles were present. Electron‐dense lysosomes were typical features of these maternal macrophages. The large ‘empty’ vesicles, which were typical for the foetal stromal macrophages, were not always observed. The maternal macrophages could be differentiated from the other main cell type in the caruncular stroma, the myofibroblasts (Figure 3). The latter were characterized by bundles of microfilaments, dense bodies and enlarged cisternae of rough endoplasmic reticulum.

### 3.2 Maternal and foetal macrophages in bovine placenta are LAMP‐1 positive

**FIGURE 4:**
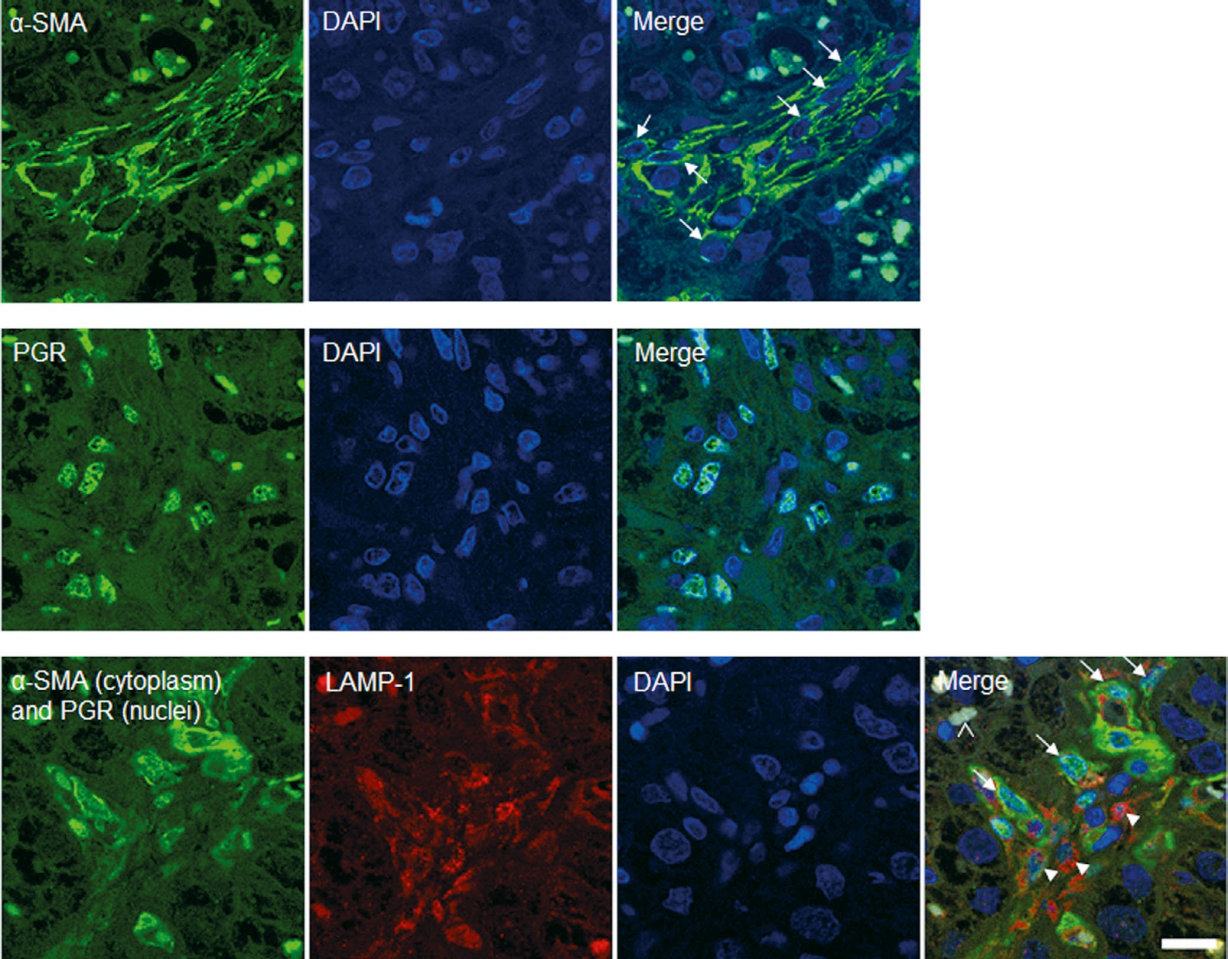
Immunofluorescent labeling of cell populations located in maternal stroma. Placental sections from healthy term cows were stained with LAMP‐1 (red), α‐SMA (green, cytoplasm) and PGR (green, nucleus). The detection of PGR was performed to more easily differentiate cell types in maternal stroma. In the merged images maternal macrophages (arrowheads) and myofibroblasts (arrows) in maternal stroma (MS) adjacent to fetal villi (FV) are labelled. Nuclei were labeled with DAPI (blue). Scale bar = 15 μm

By using three‐color fluorescence staining, we were able to distinguish two different cell populations in the caruncular stroma: cells with strong expression of α‐SMA in their cytoplasm and cells with strong cytoplasmic LAMP‐1 staining. A subpopulation of the α‐SMA positive cells showed a nuclear progesterone receptor (PGR) staining, which was absent in LAMP1‐positive cells (Figure 4).

**FIGURE 5:**
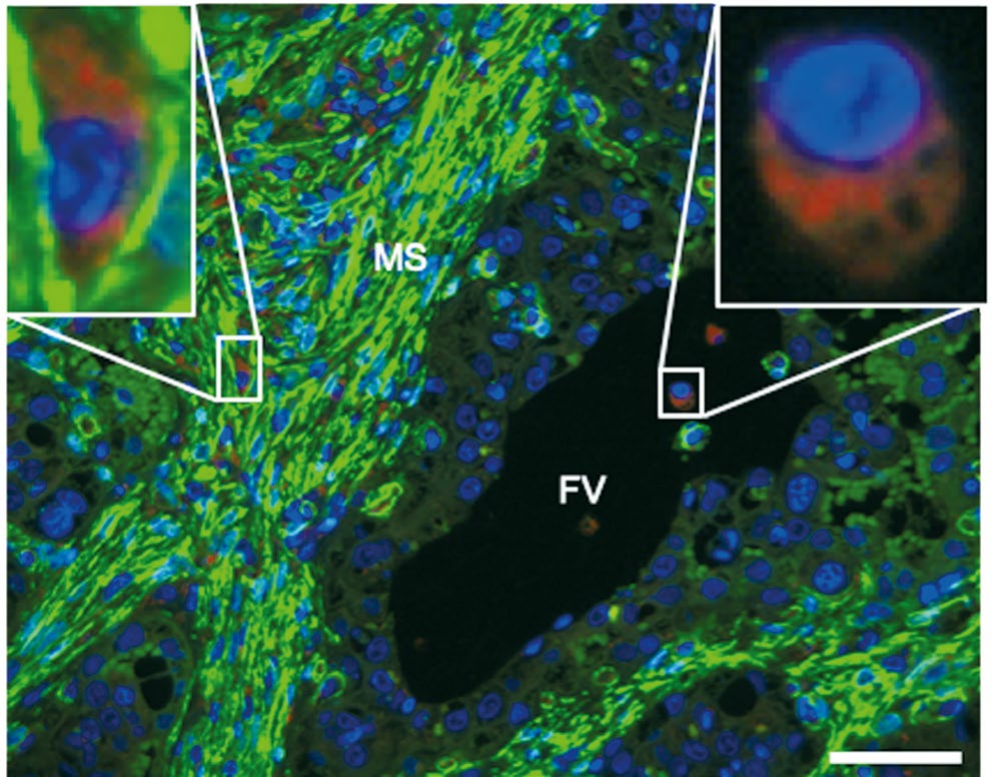
Composition and distribution of the fetal and maternal macrophage population in bovine placenta. Placental sections of a healthy term cow were stained with LAMP‐1 (red), α‐SMA (green, cytoplasm) and PGR (green, nucleus). MS: maternal stroma and FV: fetal villi. Scale bar =50μm

In the cotyledon, a few cells in foetal arteries showed positive signal against α‐SMA. Furthermore, within foetal connective tissue cells with a strong LAMP‐1 staining in their cytoplasm were detected (Figure 5). However, no signal for PGR was detected in foetal tissue. The trophoblast layer did not show any significant staining with any of the three applied antibodies (Figure 5).

In order to confirm the specificity of the α‐SMA‐ and PGR‐antibodies, which were both mouse monoclonal, we did separate staining with these antibodies. As expected, the antibodies showed cytoplasmic and nuclear binding respectively.

### 3.3 The density of foetal LAMP1‐positive macrophages showed no significant difference between control and RFM

LAMP1^+^ macrophages in foetal connective tissue were quantified and compared between the samples from normal cows and cows with RFM (20 cows/group). In cows with normal release of foetal membrane (controls), a higher density of macrophages (255.9 ± 400/mm^2^) was estimated, compared to cows with RFM (143.1 ± 128.1/mm^2^). This difference, however, was not significant (*p* = .240; Student's *t*‐test).

### 3.4 Expression pattern of the target genes is similar in maternal and foetal tissues

**FIGURE 6:**
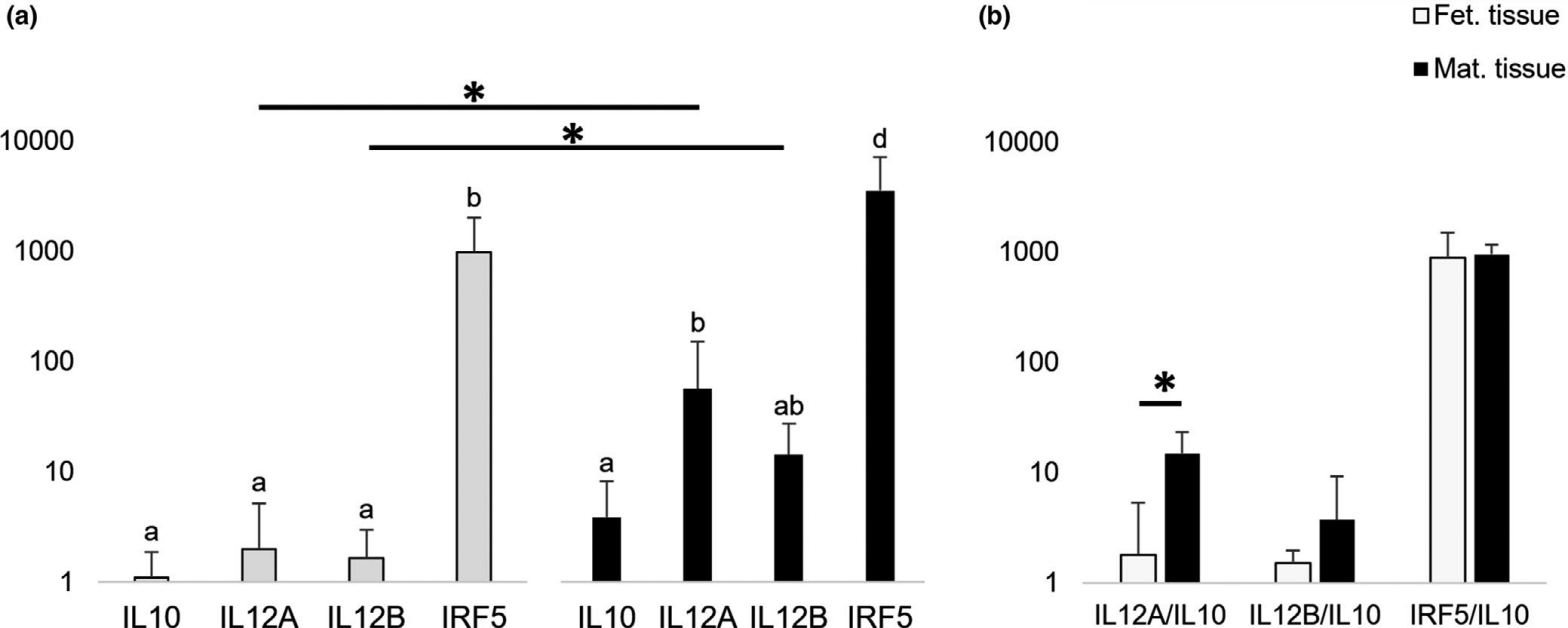
(a) Expression of IL10, IL12A, IL12B, and IRF5 as determined by Real‐Time (TaqMan) PCR. Geometric means ± geometric *SD* was compared within and between fetal and maternal compartments. Bars with different letters or star differ at *p* < .05. (b) The expression of the three M1‐polarization factors (IL12A, IL12B and IRF5) were related to the the M2‐polarization factor IL10

In both maternal and foetal tissue samples, the mRNA expression of *IL10, IL12A, IL12B and IRF5* was detectable by applying semi‐quantitative (TaqMan) PCR (Figure 6). In order to evaluate the expression of M1 (*IL12A*, *IL12B* and *IRF5*) and M2 (*IL10*) polarization markers in maternal and foetal macrophages in bovine term placenta, first their expression level within foetal and maternal tissues was analysed separately. The results showed that in both tissues, the expression of *IRF5* was significantly higher than all other genes (Figure 6). In the maternal tissue, the expression of *IL10* was significantly lower than both *IL12A* (*p* = .003) and IRF5 (*p* < .001) (Figure 6a).

Next, to compare the expression of the target genes between maternal and foetal tissues, a pairwise comparison was carried out between the two groups. Accordingly, the expression of *IL12A* and *IL12B* was significantly higher in maternal tissue (*p* = .015 and *p* = .007 respectively) (Figure 6a).

Finally, the ratio of each of M1 polarization markers and *IL10* mRNA expression levels was calculated and compared. The results indicated that the *IL12A*/*IL10* ratio was significantly higher in maternal part compared to that of in foetal tissue (*p* = .036) (Figure 6b).

## 4 DISCUSSION

The main purpose of this study was to give a better insight on the characteristics of foetal and maternal macrophages in bovine term placenta. To date, although macrophages located in the maternal compartment of the placenta have been widely studied (Mansouri‐Attia et al., 2012; Nelli et al., 2019; Oliveira and Hansen, 2009; Oliveira et al., 2010), foetal macrophages have not been investigated in detail. A successful and on‐time expulsion of the foetal membranes is associated with an appropriate inflammatory immune response at the time of parturition (Gunnink, 1984a, 1984b, 1984c, 1984d). Gradual increase of foetal macrophage number during pregnancy and even post‐partum period (Schlafer et al., 2000) suggests a possible role of macrophages in the separation and expulsion of foetal membranes. Our study shows the ultrastructural differences of foetal and maternal macrophages in bovine placenta. It further confirms the abundance of macrophages in the caruncle, coinciding with other studies (Nelli et al., 2019; Oliveira and Hansen, 2009). Transmission electron microscopy showed very different appearance of the foetal and maternal macrophages. The foetal macrophages phenotypically resemble Hofbauer cells of the human placenta (Jones et al., 2015). The large intracellular vacuoles are probably macropinosomes, which result from an uptake of the surrounding watery extracellular matrix by macropinocytosis (Lin et al., 2020). The spindle shape of the maternal macrophages in the caruncular stroma fits to their surrounding, which is much denser filled with cells and fibres. The immunolabelling for SMA and PGR revealed that the maternal and foetal macrophages are PGR‐negative and that the PGR‐positive cells in the caruncular stroma are myofibroblasts, which are also SMA‐positive. These findings are in accordance with previously published results (Greven et al., 2007) which showed PGR‐positive nuclei only in the maternal tissue. Accordingly, the authors reported specific nuclear staining predominantly in stromal cells and a few vascular pericytes, both located in caruncular septa and some small caruncular arteries.

We next examined whether the density of foetal macrophages differed between cows that expelled the foetal membranes spontaneously and those that retained them. We used a LAMP‐1 antibody for the quantification of foetal macrophages. Lamp‐1 is a lysosomal protein, which is not specific for macrophages. But this antibody gave a strong and reliable signal in the foetal and maternal macrophage, while in earlier experiments, more specific macrophage markers did not give reliable results on the paraffin‐embedded tissues (data are not shown). Together with the typical morphology of the foetal macrophages, which was observed in TEM and light microscopy, and a strong LAMP‐1 expression, we are confident that the counted cells are macrophages. No significant numerical difference of the cell density was detected between foetal macrophages in controls and in the RFM group. In controls, the mean densities of foetal macrophages were higher than in RFM animals, but due to the high variability between the individual samples, this trend was non‐significant. The high variability in the quantity of macrophages might be explained by the fact that although samples were taken at a highly precise time (within 15 min postpartum), the duration of the birth varied by several hours and consequently the distance to the initiating mechanisms of parturition (foetal cortisol increase, decrease in progesterone level, increase in Prostaglandin F2alpha availability) was not defined.

To our knowledge, this is the first study addressing the polarization status of both foetal and maternal macrophages in bovine term placenta. We compared the expression level of four genes (*IRF5, IL12A, IL12B* and *IL10*), which are involved in macrophage polarization, between foetal and maternal tissues. A high expression level of *IRF5* was detected in both parts of the placenta. As previously reported, the high expression of this transcription factor is a significant characteristic of M1 macrophages and leads to an increased expression of *IL12* and a reduced expression of *IL10* (Krausgruber et al., 2011). In addition, it has been shown that IRF5‐knockout mice produce lower levels of *IL12B* (Ouyang et al., 2007; Takaoka et al., 2005) and that the induction of *IRF5* in M2 macrophages gives rise to a higher expression of M1‐associated cytokines (Krausgruber et al., 2011). Hence, we infer that IRF5 in bovine placental macrophages functions similar to that of in human and mice macrophages. Accordingly, it is likely that abundant expression of *IRF5* in both foetal and maternal macrophages is associated with upregulation of the genes encoding *IL12* in these tissues and has suppressed the expression of *IL10*. The strong *IRF5* expression in the foetal and maternal tissues suggests that most macrophages are of the M1 type. In bovine, it has been shown that during the second half of pregnancy, the proportion of M2 macrophages increases, leading to the anti‐inflammatory milieu that is required to prevent a rejection of the foetus (Oliveira et al., 2010). However, prior to parturition, a shift in the cytokine expression towards pro‐inflammatory M1 polarization is required to promote uterine contractions and facilitate the expulsion of the calf and foetal membranes (Engelen et al., 2009). Our results on the maternal compartment of the bovine term placenta confirm the results from a previous study, asserted that during normal parturition, maternal macrophages are mostly pro‐inflammatory (Nelli et al., 2019). In a recent study (Hirayama et al., 2020), the mRNA expression of three inflammatory cytokines (*CCL2*, *CCL5*, *CCL8*), two receptors (*CCR1* and *CCR5*) and a macrophage surface marker (CD11B) has been compared between cotyledon and caruncle tissues in spontaneously delivered foetal membranes. Interestingly, the expression of all six evaluated genes is higher in caruncular tissue compared to that of the cotyledon. Together with our results, this may indicate stronger inflammatory events and more numerous macrophages in the caruncular part of the placenta. Furthermore, it supports a previous study, which showed the existence of abundant caruncular macrophages in bovine term placenta (Nelli et al., 2019). However, comparison of the *IRF5*/*IL10*, *IL12A*/*IL10* and *IL12B*/*IL10* ratios between the two groups shows that although foetal and maternal macrophages possess some different characteristics, they both promote the antepartal inflammatory milieu in bovine term placenta.

We did not possess the materials to study macrophage polarization in RFM cows. However, according to Nelli et al., (2019), caruncular macrophages in RFM samples displayed a higher expression of M2 macrophage‐associated genes (CD206, C‐type lectin domain family 7 member A (CLEC7A) and RNASE6), compared to controls. They concluded that an accumulation of M2 macrophages may prevent a sufficient infiltration of immune cells into the caruncle. This may lead to an impairment of the inflammatory, phagocytic and proteolytic processes, which are necessary for the timely release of foetal membranes.

## 5 CONCLUSIONS

The strong ultrastructural difference between foetal and maternal macrophages suggests different functions of these two cell types. Our results also indicate that at parturition, placental macrophages are mostly polarized towards the pro‐inflammatory M1 type. High expression of *IRF5* suggests a role of this transcription factor in the upregulation of M1‐specific cytokines (*IL12A* and *IL12B*). In foetal tissue, the level of macrophage‐associated transcripts was generally much lower than in the maternal tissue, which may indicate that foetal macrophages are less frequent than maternal macrophages in bovine term placenta.

## CONFLICT OF INTEREST

None of the authors have any conflict of interest to declare.

## AUTHOR CONTRIBUTIONS

RH and KK conceived the study, designed and performed the experiments, were involved in data analysis and data interpretation and wrote the manuscript. AK was involved in performing experiments, counting foetal macrophages and editing the manuscript. MPK and HZ were involved in critical discussion of data and editing of the manuscript. HZ provided tissue samples of RFM cows.

## Data Availability

The data that support the findings of this study are available from the corresponding author upon reasonable request.
